# Detection of anti-*M*. *leprae* antibodies in children in leprosy-endemic areas: A systematic review

**DOI:** 10.1371/journal.pntd.0009667

**Published:** 2021-08-27

**Authors:** Louise Pierneef, Anouk van Hooij, Anneke Taal, Raisa Rumbaut, Mauricio Lisboa Nobre, Wim van Brakel, Annemieke Geluk

**Affiliations:** 1 Dept. Infectious Diseases, Leiden University Medical Center, Leiden, The Netherlands; 2 NLR, Amsterdam, The Netherlands; 3 National Leprosy Program, Ministry of Public Health of Cuba, Havana, Cuba; 4 Giselda Trigueiro Hospital and Institute of Tropical Medicine of Rio Grande do Norte, Federal University of Rio Grande do Norte, Natal, Brazil; Johns Hopkins University, UNITED STATES

## Abstract

**Background:**

Leprosy elimination primarily targets transmission of *Mycobacterium leprae* which is not restricted to patients’ households. As interruption of transmission is imminent in many countries, a test to detect infected asymptomatic individuals who can perpetuate transmission is required. Antibodies directed against *M*. *leprae* antigens are indicative of *M*. *leprae* infection but cannot discriminate between active and past infection. Seroprevalence in young children, however, reflects recent *M*. *leprae* infection and may thus be used to monitor transmission in an area. Therefore, this literature review aimed to evaluate what has been reported on serological tests measuring anti-*M*. *leprae* antibodies in children without leprosy below the age of 15 in leprosy-endemic areas.

**Methods and findings:**

A literature search was performed in the databases Pubmed, Infolep, Web of Science and The Virtual Health Library. From the 724 articles identified through the search criteria, 28 full-text articles fulfilled all inclusion criteria. Two additional papers were identified through snowballing, resulting in a total of 30 articles reporting data from ten countries. All serological tests measured antibodies against phenolic glycolipid-I or synthetic derivatives thereof, either quantitatively (ELISA or UCP-LFA) or qualitatively (ML-flow or NDO-LID rapid test). The median seroprevalence in children in endemic areas was 14.9% and was stable over time if disease incidence remained unchanged. Importantly, seroprevalence decreased with age, indicating that children are a suitable group for sensitive assessment of recent *M*. *leprae* infection. However, direct comparison between areas, solely based on the data reported in these studies, was impeded by the use of different tests and variable cut-off levels.

**Conclusions:**

Quantitative anti-PGL-I serology in young children holds promise as a screening test to assess *M*. *leprae* infection and may be applied as a proxy for transmission and thereby as a means to monitor the effect of (prophylactic) interventions on the route to leprosy elimination.

## Author summary

Leprosy, a chronic infectious disease caused by *Mycobacterium leprae (M*. *leprae)*, targets the skin and nerves and often results in irreversible disabilities as well as social exclusion. Though the disease can be efficiently treated, leprosy elimination is hampered by ongoing transmission of *M*. *leprae*. Currently, elimination is monitored by the number of new cases. Since only a small percentage of individuals infected with *M*. *leprae* develops disease, this does not accommodate monitoring of transmission. Previous studies have shown that antibody levels against *M*. *leprae* in blood correspond to the bacterial load in an individual and can be used as a proxy for infection, although antibodies cannot distinguish between past and present infection. In young children, infection is recent by definition, which allows assessment of more current state of transmission in an area. Thus, this literature review investigated studies on leprosy serology in children without leprosy. Our findings underscore that young children are a fitting group for up-to-date monitoring of *M*. *leprae* transmission in an area as seropositivity is inversely related with age. Importantly, a standardized, field-friendly test quantitatively measuring anti-*M*. *leprae* antibodies should be applied for population screening to monitor the status of transmission and thereby elimination in an area.

## Introduction

Leprosy is a neglected tropical disease caused by *Mycobacterium leprae (M*. *leprae)* or *M*. *lepromatosis* with tropism for skin and peripheral nerves often leading to skin lesions, loss of sensation and nerve damage that cause lifelong physical and social disabilities [1,2]. It afflicts marginalized populations in low- and middle-income countries (LMICs) in their most productive stage of life [3–5] and despite the availability of effective treatment still poses a public health problem [6]. The main route of *M*. *leprae* transmission is generally considered to be from human to human via aerosol droplets spread by the respiratory route [7]. However, in general, frequent and high dose exposure to *M*. *leprae* is thought to be required for development of disease. Consequently, household members of untreated leprosy patients are at higher risk of contracting leprosy [8–10].

Despite decades of control efforts using multidrug therapy (MDT), the global number of new leprosy cases has remained stable for over a decade just above 200,000 annually [11]. More than 15,000 new cases are found each year among children below 15 years of age, indicating continuous and recent *M*. *leprae* transmission in many endemic communities. On top of that, expertise of health care professionals to recognize signs and symptoms of leprosy, particularly at early stages, has declined [12]. This often results in delayed diagnosis, which increases the risk of permanent disabilities and other consequences [13–17].

In order to achieve elimination of leprosy, strategies involving early diagnosis through active case finding and contact tracing combined with postexposure prophylactic (PEP) treatment [18–23] are essential to interrupt transmission and prevent development of leprosy in high-risk contacts [24,25].

In response to the current status of leprosy health care and the stable number of new cases, the Global Leprosy Strategy 2021–2030 has set a target of 90% reduction in rate per million of new cases with grade 2 disability (G2D) as well as 90% reduction in rate of new child cases with leprosy [26], thus combining activities promoting early detection of leprosy and reduction of transmission. Current (programme) indicators used by the WHO to monitor the progress of elimination of leprosy are based on the proportion of child cases (below 15 years of age) among total new cases detected [27]. However, as the incubation period of leprosy is long, on average 5 years, but often much longer, utilizing a time span of 15 years does not sensitively reflect recent transmission. In addition, only a small percentage (estimated 5%) of individuals infected with *M*. *leprae* actually develop disease [1] which renders use of new leprosy cases, as used in the current indicators for leprosy elimination [27], unsuitable for determination of *M*. *leprae* transmission in an area, stressing the need for an alternative indicator.

It has been amply described that levels of IgM antibodies against *M*. *leprae* phenolic glycolipid I (PGL-I) correlate strongly with the bacterial load within individuals [28–30] and animals [31]. Moreover, these antibodies are reduced upon efficient treatment of patients [32–34]. These findings have generated the concept of serological tests that detect antibodies against *M*. *leprae* as an indicator of the proportion of the population that has been infected. This includes asymptomatic infection, which is overlooked if only patients are considered [35–38]. Although antibody levels shed some light on the infection status [28,33], their presence cannot discriminate sufficiently between recent and past infection [39]. However, finding evidence of infection in (young) children would by definition indicate recent transmission. Therefore, measuring seroprevalence among young children represents a potential tool to monitor the intensity of recent transmission, thereby informing on the elimination of transmission. Particularly when antibody levels are assessed quantitatively [28], monitoring longitudinal changes in seroprevalence measured cross-sectionally among young children of a certain age group is indicative of the status of transmission as well as the effect of control measures taken in an area [38,40].

We conducted a systematic literature review to determine what has been investigated with respect to screening of anti-*M*. *leprae* antibodies in children without leprosy and known contact with patients diagnosed with leprosy.

## Methods

**Fig 1:**
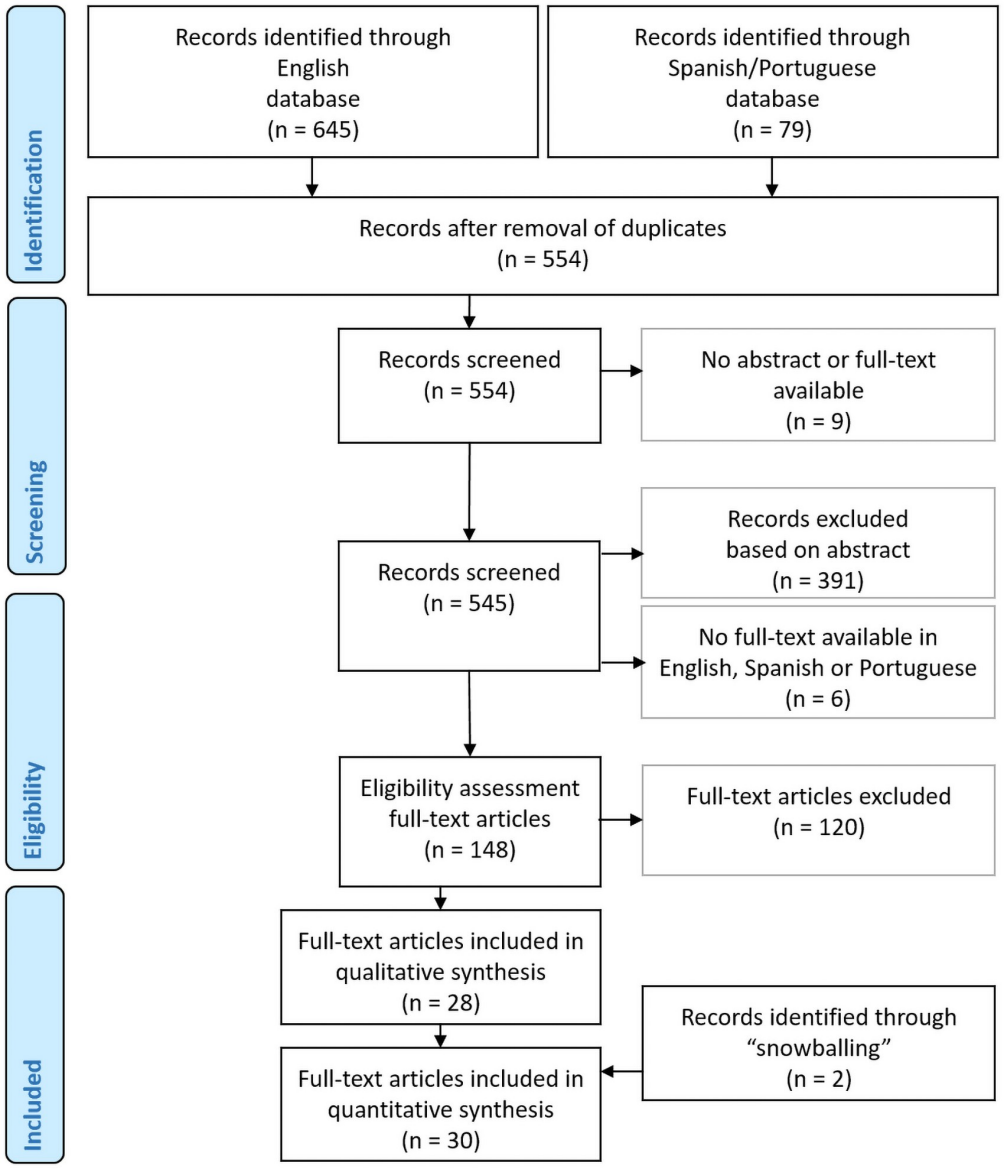
PRISMA flow chart. Overview of the selection procedure of 30 full-text articles included in the review.

A systematic review was conducted according to the recommendations of the Preferred Reporting Items for Systematic reviews and Meta-Analyses (PRISMA) [41], targeting leprosy serology in children using PubMed (https://pubmed.ncbi.nlm.nih.gov/), Web of Science (www.webofknowledge.com), Infolep (https://www.leprosy-information.org/) and The Virtual Health Library (VHL; https://pesquisa.bvsalud.org/portal/) as sources. The search strategy shown in the PRISMA flowchart (Fig 1) using the search strings listed in Table 1, included all available peer-reviewed publications until July 1, 2020. If no full-text was available in the databases described above, full-texts were found in one of the following online libraries: Europepmc (https://europepmc.org/), ILSL http://www.ilsl.br/), J-STAGE (https://www.jstage.jst.go.jp/), Oxford Academic (https://academic.oup.com/journals), Researchgate (https://www.researchgate.net/), SAGE journals (https://journals.sagepub.com), Scielo (https://scielo.org/en/), Sciencedirect (https://www.sciencedirect.com/), Semantic Scholar (https://www.semanticscholar.org/), Taylor & Francis online (https://www.tandfonline.com/), the WHO website (https://www.who.int/), Wiley Online (https://onlinelibrary.wiley.com/) and obtained from the Royal Library (Koninklijke Bibliotheek) (https://www.kb.nl/) in The Hague, the Netherlands. Articles identified through the English databases were screened for eligibility based on the title, abstract, and finally on a full-text assessment by three authors (LP, AT and AG). The resulting lists were compared and a consensus reached on eligibility of the articles for selection. Additionally, the reference lists of the included articles were manually scrutinized for primary studies that could have been lost in the electronic search (“snowballing”). Articles in Portuguese or Spanish were screened for eligibility by two native speakers (MLN and RR). The authors were not blinded to the names of the study authors, journals or institutions.

**Table 1:** Search strings applied per database.

Database	Hyperlink	Search string
Pubmed	https://pubmed.ncbi.nlm.nih.gov/	(leprosy AND schoolchildren AND detection) OR (leprosy AND (children OR adolescent) AND (serum OR serology))
Web of Science	www.webofknowledge.com	ALL = (leprosy AND schoolchildren AND detection) OR ALL = (leprosy AND (children OR adolescent) AND (serum OR serology))
Infolep	https://www.leprosy-information.org/	“leprosy” “children” “serology” “leprosy” “children” “serum” “leprosy” “adolescent” “serology” “leprosy” “schoolchildren” “detection”
The Virtual Health Library	https://pesquisa.bvsalud.org/portal/	(leprosy AND schoolchildren AND detection) OR (leprosy AND (children OR adolescent) AND (serum OR serology)) (lepra AND escolares AND detección) OR (lepra AND (niños OR adolescentes) AND (serología)

This table shows the hyperlinks and search strings used for Pubmed, Web of Science, Infolep and The Virtual Health Library.

### Inclusion and exclusion criteria

Articles were included if they described original studies on leprosy serology in children below 15 years of age who were not affected by leprosy. Articles were excluded if they primarily discussed studies on (neglected tropical) diseases other than leprosy, reported studies on serology related only to leprosy disease or to leprosy reactions, or if they lacked critical information on the serological methods applied or the number of the children recruited. Articles were also excluded if the abstract in English fulfilled the inclusion criteria but the rest of the text was not available in English, Spanish, or Portuguese.

### Data extraction and analysis

After inclusion of eligible papers, information about the study design, the type of serological test used (including the (laboratory) protocol and target antigen), the year, the research location, the study population, the age or age group, gender, the sample size of children tested and the seropositivity percentage(s) reported were extracted into Microsoft Excel 2016. Data extraction was done in duplicate (LP and AT for English publications; MLN and RR for Portuguese and Spanish publications). If reported, the sampling and selection method for the inclusion of participants was recorded. Graphs and figures were created in GraphPad Prism 8.4.2 and Microsoft Powerpoint 2016.

Quantitative data was copied from the full texts. In case percentages for seropositivity in children were not available or could not be derived from the text, they were calculated or estimated from figures in the articles.

The median of the seroprevalence reported was calculated in Microsoft Excel 2016 by including all seropositivity percentages reported. In case multiple seropositivity percentages were reported in one article, those were included in line with how they were presented e.g., by district, by age group, or by test and depicted separately. The IQR was determined as the difference between the third and first quartile when the set of data was divided into four equal portions.

Pearson correlation method in Graphpad Prism 8 (version 8.4.2) was used for comparison of anti-PGL-I ELISA data (OD_450_) of 58 serum samples of children below the age of 15, which were previously assessed in two different studies [28,42] using ELISAs with distinct cut-off values. These cut-offs were either based on ODs corrected for background OD values that were higher than 0.2 [28] or OD values not corrected for background, but higher than the average plus three times the standard deviation of the test results from 14 healthy subjects from the same hyperendemic area (0.295) [42].

No formal methods for assessing risk of bias were used due to the variation of study methods. Study limitations influencing outcomes and conclusions were considered and described in the Discussion section of this review. For the PRISMA checklist we refer to S1 Table.

## Results

Applying the search strategy described above, a total of 724 articles were identified from the major electronic databases utilized (Fig 1). After removing 170 duplicates, 554 articles were screened for the inclusion criteria of this review. Nine articles were excluded as only a title was available, while the abstract and full-text were lacking. Subsequently, 391 additional articles were excluded based on the abstracts which indicated the lack of data on leprosy and/or serology and/or children without leprosy. Six English abstracts (of which one article in Bahasa, Indonesia) fulfilled the inclusion criteria, but no full-text was available in English, Spanish or Portuguese. For the remaining 148 articles, a full-text screening was performed, resulting in the exclusion of another 120 articles according to the selection criteria. Snowballing yielded an additional two articles. Therefore, the final selection included in this review consisted of 30 full-text articles; 27 in English and three in Portuguese.

### Participant characteristics

**Fig 2:**
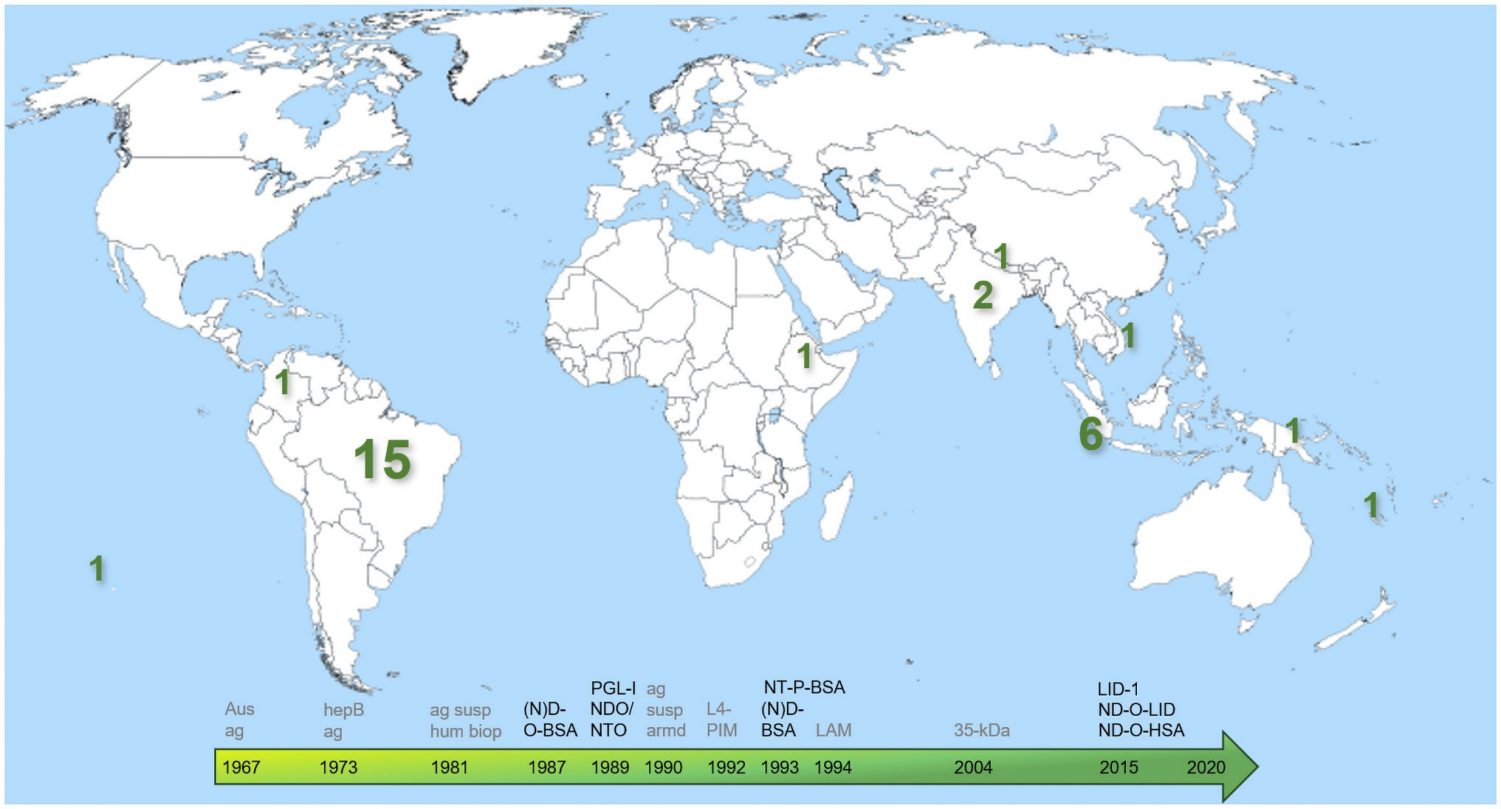
Leprosy serology in children without leprosy. Schematic representation of the countries where the selected studies in this review were located. The green numbers indicate the number of studies per country. The arrow indicates the chronological use of target antigens applied for leprosy serology. The antigens colored in grey, are those used in studies reporting leprosy serology among children without leprosy, which did not fulfil all inclusion criteria of this review. Aus ag: Australia antigen; hepB ag: hepatitis B antigen; ag susp hum biop: antigen suspension prepared from human leprosy biopsy tissue; ag susp arm: suspension of *M*. *leprae* purified from an infected armadillo’s liver; (N)D-O-BSA: (natural) disaccharide-octyl bovine serum albumin; PGL-I: phenolic glycolipid I; NTO: natural trisaccharide-octyl; L4-PIM: L4-reactive antigen from *M*. *tuberculosis*; NT-P-BSA: natural trisaccharide phenylpropionyl bovine serum albumin; LAM: lipoarabinomannan; 35-kDa: 35-kilodalton protein of *M*. *tuberculosis*; LID-1: leprosy IDRI diagnostic-1; ND-O-LID: semi-synthetic disaccharide attached to the octyl radical, which mimics PGL-I, conjugated with two fusion proteins, ML0304 and ML0331, forming LID; ND-O-HSA: natural disaccharide-octyl human serum albumin. The template for this world map was retrieved from: https://www.worldofmaps.net/weltkarten/weltkarten-und-weltatlas/weltkarte-blank-vektorgrafik.htm. Colored numbers and a timeline were added.

The data presented in the selected articles covered more than 18,000 children across ten countries studied between 1987 and 2020 (Table 2 and Fig 2). Half of the studies were conducted in Brazil (n = 15). Children included household contacts of persons affected by leprosy, non-household contacts, schoolchildren and residents from regions considered high-, medium-, low- and non-endemic for leprosy, who were not known to be affected by leprosy. For most studies, sampling methods were not reported. The results on the prevalence of seropositivity for *M*. *leprae* in children found in the included articles are summarized in Table 3 and S2 Table and S1 Fig. For each area, results are shown separately for children with and without known contact with leprosy patients. The median seroprevalence in children in the study areas included was 14.9% (IQR = 7.3–27.7).

**Table 2:** Publications on leprosy serology in children without leprosy from ten countries.

Country	Publications	# of children	Publication date (range)
Brazil	15	10,423	2008–2019
Colombia	1	49	2008
Ethiopia	1	40	1987
French Polynesia	1	~20*	1993
India	2	3,449	1991–1994
Indonesia	6	~3,880*	1994–2020
Nepal	1	93	1994
New Caledonia	1	15	1989
Papua New Guinea	1	62	1990
Vietnam	1	342	2018
**Total**	**30**	**~18,373**	**1987–2020**

This table shows the sum of the number of children that were reported in the included studies listed per country.

*Estimated from figure.

**Table 3:** Prevalence of seropositivity for *M*. *leprae* antibodies in children without leprosy sorted per country and test group.

Country (area)	Ref. no.	Author	Year of publication	Applied methods						NCDR (per 100,000) per area	Seropositivity (%) for:	
				ELISA	UCP-LFA	ML-dipstick	ML-flow test	NDO-LID rapid test	GPAT		(endemic) controls	contacts
Brazil (Pará)	42	Barreto	2011	yes							ranged from 57.4 to 63.6	44.1
Brazil (Pará)	43	Barreto	2015	yes							77.6	
Brazil (Pará)	45	van Hooij	2018		yes						6.0	
Brazil (South(east))	36	Bührer-Sékula	2008	yes		yes				Governador Valadares: 112	18.6 / 14.2	
										Santa Luzia: 10	23.0 / -	
										Barbacena: 2	15.0 / 12.8	
										Aracruz: 37	24.0 / 14.4	
										Colatina: 35	14.0 / 10.1	
										Santa Teresa: 22	15.0 / 17.4	
										Itajaí: 9	16.9 / 14.2	
										Laguna: 5	12.0 / 12.5	
										Tubarão: 5	12.0 / 8.5	
Brazil (South(east))	46	da Conceição	2011	yes							1.4	
Brazil (South(east))	47	Ribeiro	2019	yes							10.1	
Brazil (South(east))	48	Ferreira	2008				yes					12.3
Brazil (South(east))	49	Andrade	2008				yes					17.8
Brazil (South(east))	50	Carvalho	2015				yes					18.6
Brazil (South(east))	51	Wambier	2016	yes								18.0
Brazil (South(east))	52	Dúppre	2008			yes	yes					14.7
Brazil (Midwest)	53	Limeira	2013				yes					0.0
Brazil (Midwest)	54	Gomes	2019					yes				6.2
Brazil (Northeast)	55	Lourenço	2017				yes					33.7
Brazil (Northeast)	56	TiemiNagao-Dias	2019	yes								IgM: ranged from 40.0 to 46.7
				yes								IgA: ranged from 0.0 to 4.4
				yes								IgG: ranged from 0.0 to 2.2
Colombia	57	Cardona-Castro	2008	yes								51.0
New Caledonia	58	Desforges	1989	yes								NDO: 26.7
												NTO: 13.3
French Polynesia	59	Chanteau	1993	yes								ranged from ~18.0 to 23.0*
Papua New Guinea	61	Bagshawe	1990	yes							ranged from 21.0 to 30.6	
Ethiopia	62	Menzel	1987	yes							35.7	50.0
Nepal	63	Soares	1994	yes								1.1
India (South)	64	Krishnamurthy	1991	yes							ranged from 5.14 to 16.74	ranged from 6.98 to 28.13
India	65	Shah	1994	yes							LER: 0.0	
											HER: 6.9	
												21.4
Vietnam	66	Khang	2018						yes		2.5**	17.0
Indonesia (South Sulawesi)	68	van Beers	1994						yes		ranged from ~35.0 to 37.0*	
				yes							IgM: ranged from ~37.0 to 40.0*	
				yes							IgG: ~6.0*	
Indonesia (Sulawesi)	35	v an Beers	1999						yes	Kauditan: 43	26.0	
										Bantimurung: 41	28.0	
										Bontomarannu: 34	26.0	
										Lawa: 6	7.0	
										Tongkuno: 9	7.0	
Indonesia (South Sulawesi)	73	Bakker	2005	yes							14.3	
Indonesia (South Sulawesi)	74	Putri	2010	yes							ranged from 39.7 to 48.3	
Indonesia (South Sulawesi)	75	Rachmawati	2013	yes							ranged from 40.0 to 57.5	
Indonesia (East Java)	76	Adriaty	2020	yes							non-endemic: 8.3**	
											endemic: 48.0	

Overview of the prevalence of seropositivity for anti-*M*. *leprae* antibodies in children without leprosy sorted per country and test group (endemic controls or contacts of leprosy patients). For each study, the country, reference number, author, year of publication, laboratory assay and seroprevalence (%) for *M*. *leprae* antibodies are reported for either (endemic) controls or (household) contacts of leprosy patients. For the numbers of children included and the method used per study, we refer to the Supplementary. The boxes in grey indicate that this data was not available. The antibody subtypes measured are indicated by the colors in the applied methods column; green: immunoglobulin M (IgM), blue: immunoglobulin A (IgA), yellow: immunoglobulin G (IgG), orange: both IgM and IgG and purple: not reported.

*Estimated from graph/figure.

**Reportedly leprosy non-endemic area.

CP: contact population from a leprosy colony; ELISA: enzyme-linked immunosorbent assay; F: females; GPAT: gelatin particle agglutination test; HER: children from reportedly high-endemic region; LER: children from reportedly low-endemic region; M: males; NCDR: new case detection rate; NDO: natural disaccharide-octyl; NTO: natural trisaccharide-octyl; ref. no.: reference number; UCP-LFA: up-converting phosphor lateral flow assay.

### Latin America

Pará has a high annual leprosy case detection rate of 50/100,000 persons in comparison to the lower average of 17/100,000 in Brazil (data from 2012) [43]. From 2011 to 2015, Barreto and Salgado and colleagues carried out several studies in the Brazilian Amazon region on leprosy seroprevalence including children [42–44] (Table 3 and S2 and S3 Tables). Seropositivity in their studies was determined by ELISA and defined as all values above the mean optical density (OD) plus three standard deviations (from sera from 14 healthy subjects from the same hyperendemic area). In 2011, they found proportions of 63.3% and 57.4% seropositive for antibodies against PGL-I in a cross-sectional study among random samples of 49 and 47 schoolchildren from two elementary schools in Castanhal, state of Pará, Brazil [42]. No correlation was found between age of the children and seroprevalence. Based on the high seroprevalence rates, the authors assumed that *M*. *leprae* is circulating actively among this population. In 2015 [43], these researchers identified a similar seroprevalence rate (77.6%) among 134 schoolchildren. Child contacts of leprosy patients (44.1% of 68 children) did not show higher seroprevalence [42].

In contrast to the high percentages above, only 6% seropositivity was reported by van Hooij *et al*., measuring anti-PGL-I IgM levels using the up-converting phosphor lateral flow assay (UCP-LFA) [28,45] in sera of 207 schoolchildren from Pará without known contact with leprosy patients. As the variety of serological tests used in the selected studies impede direct comparison of results, we performed a direct comparison of the serology data of children from the same region in Pará, obtained by anti-PGL-I IgM ELISAs of both research groups (n = 58) which applied a different cut-off, but showed excellent correlation (p<0.0001; Pearson r = 0.92; S2A Fig). Focussing subsequently into the twenty samples that had values around the cut-off value (S2B Fig), nine of these scored low positive in the assay performed by Barreto *et al*. [42], whereas only one scored positive in the ELISA by van Hooij *et al*. [28] (S2C Fig). This emphasizes the importance of not only the type of serological test, but also the cut-off used in seroscreening for proper assessment of transmission.

In South(east) Brazil [36], 7,073 schoolchildren were assessed in three leprosy-endemic states Espírito Santo (ES), Minas Gerais (MG) and Santa Catarina (SC), with a leprosy incidence of 41, 16 and 4 per 100,000, respectively (1998) using both quantitative anti-PGL-I ELISA and qualitative ML-dipstick (Table 3 and S2 Table). In all areas, seropositivity levels ranged from 8.5% to 24%, and there was no clear correlation found between leprosy detection rates and seropositivity rates. In addition, in two other areas in Minas Gerais seroprevalence rates of 1.4% (2011; n = 355) [46] and 10.1% (2019; n = 358) among schoolchildren were found [47].

In the same state, seropositivity percentages (ML-flow tests) among child household contacts ranged from 12.3% to 18.6% [48–50]. In São Paulo state, 18% of child household contacts tested seropositive (ELISA) [51] whereas this was the case in 14.7% in Rio de Janeiro (ML-dipstick/ML-flow) [52].

Besides test type and cut-off, also the sample size needs to be taken into consideration when comparing seropositivity data as demonstrated by the differences in seropositivity identified in a very small study in Midwest Brazil (n = 15; 0% positivity) [53], and 4 to 6.9% among 210 contacts in another study from the same area (Table 3) [54].

In contrast, in Northeast Brazil, higher seropositivity rates were found among household and neighborhood contacts under 15 years of age; 33.7% in Ceará [55] and 40% to 46.7% (IgM) in Alagoas were seropositive [56]. However, seropositivity for IgA (0 to 6.6%) and IgG (0 to 2.2%) was very low (Table 3).

In Colombia, a cross-sectional survey among household child contacts in 13 towns located in Bolívar, Córdoba and Sucre, Colombia [57] (Table 3) found that 25 out of 49 (51%) contacts had positive anti-PGL-I IgM titers. No association was found between IgM positivity and clinical diagnosis of the patients.

### The South-Pacific

In 1989, serological screening was conducted among household contacts of leprosy patients (n = 15) in New Caledonia using ELISAs for two synthetic forms of PGL-I [58]. For ND-O-BSA, 26.7% and for NT-O-BSA, 13.3% of the contacts was positive (Table 3). Although the number of children in the study was limited, a trend was observed that seropositivity was higher in individuals below the age of 20 (compared to participants over the age of 20) and inversely decreased with age, which led the authors to conclude that the transmission rate in younger individuals is higher. Furthermore, ND-O-BSA was considered to be more sensitive, and NT-O-BSA more specific for screening for *M*. *leprae* infection among household contacts.

A 10-year study in French Polynesia [59] also based on anti-PGL-I ELISAs showed that of family contacts of patients diagnosed with leprosy (n = 20; note that this sample size is very small), approximately 18% of the boys and about 23% of the girls were seropositive (Table 3). Of note is that in individuals over the age of 19, seropositivity started to decrease with age. Although some adult participants were followed up, no longitudinal results were provided for children. The authors conclude that the predictive value of positivity for anti-PGL-I is low, as most of the seropositive participants did not progress to disease during the study period. A finding which was confirmed years later in Bangladesh as well [60].

A two-year follow-up study measured serum anti-PGL-I levels in residents from Kalo village, Papua New Guinea, in 1984 and 1986, among 62 children [61] (Table 3). In 1984, 13 out of 62 (21%) children were seropositive and in 1986, this was the case for 19 out of 62 (30.6%). Similar to what was found in New Caledonia, seropositivity among participants below 20 years of age was significantly higher (20.6% among 379 individuals) compared to that among older participants (7%; *p* < 0.001).

### Africa

In 1987 in Ethiopia, Menzel *et al*. studied child household contacts of leprosy patients while also taking into account the index cases’ leprosy type [62]. Six out of 12 (50%) children with 1 or more years of exposure to a lepromatous patient were positive in anti-PGL-I ELISAs versus 10 out of 28 (35.7%) amongst endemic controls (Table 3).

### Asia

In Nepal, the use of anti-PGL-I serology by ELISA for early diagnosis among close child contacts from a reportedly low-endemic area was evaluated in 1994 [63]. Only one out of 93 children (1.1%) tested positive, and none of the contacts developed clinical disease after 6 months.

Our literature search resulted in two studies from India. The first study in 1991 in an endemic population from South India [64] included 179 child contacts and 1,564 non-contacts using cluster sampling. Contacts included children who had stayed for at least half a year with the index case. Forty-eight contacts aged 0 to 4, 75 contacts aged 5 to 9 and 56 contacts aged 10 to 14 were included. The non-contact population consisted of 467 children aged 0 to 4, 586 children aged 5 to 9 and 511 children aged 10 to 14. Seroprevalence among contacts was higher in girls (28.1%; boys: 12.5%), whereas in non-contacts boys and girls showed similar seroprevalence (S2 Table). In 1994 [65], using ELISAs children living in a reportedly low-endemic region (LER; 71 children), a high-endemic region (HER; 1495 children)—Mumbai—and a contact population (CP; 140 children) comprising household contacts of leprosy patients living in a leprosy colony were assessed. Seropositivity among these children ranged from 0% in the low endemic region, 6.9% in the high endemic area to 21.4% in the contact population (Table 3).

A divergent leprosy-specific serological test, the gelatin particle agglutination test (GPAT), has been applied by Khang *et al*. in Vietnam from 1989 to 2016 to screen 78 healthy child controls (<15) without known contact with leprosy patients living in reportedly leprosy-exempt areas and 264 household child contacts [66]. This test, based on semi-synthetic trisaccharides antigen (NT-P-BSA), was positive in 2.5% of controls and 17% of contacts (Table 3). Interestingly, there was no significant difference in seroprevalence between children and adults.

Most of the studies reporting on serum anti-PGL-I antibodies in children from Indonesia took place in Sulawesi, which in the 90s was an area with one of the highest prevalences of leprosy in Indonesia [67]. In 1994, van Beers *et al*. investigated household child contacts of leprosy cases and non-contacts in the villages Bantimala and Tondongkura for their serum anti-PGL-I antibody levels as well as the presence of *M*. *leprae* DNA in their nasal cavities [68]. Although serum anti-PGL-I IgM antibodies are studied most frequently and are in general more often positive [40,56,69–72], van Beers *et al*. also evaluated anti-PGL-I IgG in approximately 370 children. An agglutination test (MLPA; IgM) was used as well as an ELISA (IgM and IgG) with NT-P-BSA as target antigen. As seropositivity rates did not differ between household contacts and non-contacts, results were combined. Seropositivity for MLPA and anti-PGL-I IgM was highest in the younger age groups and reduced with increasing age (test agreement of 91%). In contrast, anti-PGL-I IgG seropositivity was low in all age groups. Comparison of PCR-test results (detecting bacterial DNA) with serological test outcome did not show a good correlation. According to the authors, this is not surprising as nasal carriage does not necessarily imply infection [30].

Five years later, the same researchers conducted a study in Sulawesi [35] to assess whether seroprevalence among schoolchildren included by cluster sampling could serve as a valid marker of leprosy endemicity. In this study, they included 2,835 schoolchildren from South, North and Southeast Sulawesi, with case detection rates of 39, 18 and 9.9 per 100,000, respectively. For areas with case detection rates over 30 in 100,000, seroprevalence ranged from 26 to 28%, whereas for reportedly low-endemic areas, seroprevalence was approximately 7% using MLPA. In Bantimurung, considered high-endemic in this study, a second survey was performed three years later among 905 children yielding the same seroprevalence rate of 28%. Based on their findings the authors concluded that seropositivity is related to the incidence of leprosy thus representing a rapid, reliable and relatively inexpensive approach to estimate the leprosy burden in a certain region. Another study reporting data from South Sulawesi (the island of Kembanglemari, a high-endemic area) by Bakker *et al*. [73], measured anti-PGL-I levels of inhabitants by ELISA. In the age group from 0 to 5, one out of seven individuals (14.3%) was seropositive for anti-PGL-I IgM. Clinical disease was more prevalent in adults while seropositivity was higher in children. Longitudinal investigation (2007–2010) in schoolchildren in South Sulawesi [74] detected 39.7% seropositivity (23 out of 58) in 2007, which increased three years later to 48.3% (28 out of 58 children). None of the 23 seropositive children showed clinical signs of leprosy in 2010, and 22 of them remained seropositive. Also, mean titers of anti-PGL-I IgM significantly increased from 2007 to 2010. Another study on schoolchildren in 2013, compared anti-PGL-I IgM levels and PCR results from nasal swabs in South Sulawesi [75] for 80 schoolchildren. Twenty-three out of 40 children (57.5%) from lowland were seropositive compared to 16 out of 40 (40%) from highland. Only one child in both low- and highland tested positive by PCR (2.5%). Most of the seropositive children were not known to have any contact history with leprosy patients and the authors suggested that infection may have originated from unidentified “back-log cases”, from non-human *M*. *leprae* sources in the environment or from travelling to surrounding areas. A recent paper from 2020 analyzed the presence of *M*. *leprae* in nasal swabs and seropositivity levels among 530 elementary schoolchildren from the northern and southern coast of East Java [76]. In Pacitan, an area considered non-endemic for leprosy, 25 out of 301 children (8.3%) were seropositive and in Lamongan, an area considered endemic for leprosy, seropositivity was 48% (110 out of 229). PCR results showed 23 out of 229 nasal swabs from children to contain *M*. *leprae* DNA in Lamongan (10%) in contrast to Pacitan, with only six positive nasal swab samples from a total of 301 (2%). It was calculated that children living in endemic areas were approximately five times more likely to become infected than children living in non-endemic areas.

### Cross-sectional studies in time

As fluctuations in seropositivity in time can provide information on changes in transmission in a certain area, the changes in seroprevalence among children without leprosy in the ten longitudinal studies included in the selected articles were considered (S3 Table): nine of these followed the same children over time to investigate the assessment of anti-PGL-I as a predictive marker for leprosy [43,53,56,58,59,61,63,66,74]. The remaining study measured seropositivity cross-sectionally at two time points with a 3-year interval and validated the use of anti-PGL-I seroprevalence in schoolchildren in Sulawesi (n = 2,835; 10 to 12-year-olds) as an indicator for the level of leprosy endemicity [35]. In the study of van Beers *et al*., the percentage of seropositivity (28%), as well as the leprosy case detection rate (41/100,000) in the area remained similar over three years [35].

## Discussion

### Relevance of anti-*M*. *leprae* antibody detection for monitoring transmission of leprosy

Accurate knowledge on transmission in an area can provide insight into the effect of interventions as well as enable monitoring of the transmission level towards interruption of transmission. Monitoring transmission using disease incidence implies a delay in describing the status of transmission. This is particularly the case for leprosy due to its incubation time of typically three to five years, which can, however, also exceed ten years. For this reason, the incidence of leprosy in children under 15 –the normal definition used in leprosy statistics to date–is not very sensitive to changes in transmission as older children may have been infected ten or more years ago. In contrast, monitoring of *M*. *leprae* infection in young children, represents a more up-to-date view on the status of transmission in an area.

This systematic review assessed 30 scientific articles reporting on *M*. *leprae* serology in children under 15 years evaluating to what extent and in what context children have been assessed for the presence of anti-*M*. *leprae* antibodies as a proxy for infection. We aimed to investigate whether seroprevalence among children could serve as a reliable indicator for the intensity of recent transmission in a community.

### Factors influencing seroprevalence

The 30 included articles showed that seroprevalence in over 18,000 children from over ten countries varied from 0 to 77.6% with a median value of 14.9% (IQR: 7.3–27.7). Children often were only a subgroup of a larger study also including other age groups.

Importantly, six independent studies using different serological tests conducted in (South)eastern Brazil reported similar seropositivity percentages in the range of 10.1 to 18.6% [47–52]. Surprisingly, seroprevalence rates did not differ significantly between household contacts and non-contacts. This may be related to the fact that most studies were done in (highly) endemic areas in which other sources than contact cases have been reported to play a role in transmission [77].

Interestingly, multiple studies (also including adults) [58,59,61,68,73] observed higher positivity rates in the younger age groups and a decrease in seropositivity inversely related with age, a finding which was already described 30 years ago by Ulrich *et al*. in Venezuela [78]. According to Menzel *et al*., this underlines the good sensitizing capacity of the immune system present at a young age [62]. Thus, children can be considered a suitable group for sensitive assessment of recent *M*. *leprae* infection. Fine *et al*. studied anti-PGL-I antibody seroprevalence among children in Northern Malawi [79], in a paper excluded during the screening process of this review as the numbers of the children included were not reported. In agreement with what was described above, this study in Malawi described that the seroprevalence rate peaks between the ages of 20 and 30 and then drops. Regardless of age, they also found consistently higher seropositivity rates among females compared to males. In contrast, Andrade *et al*. found positivity to be significantly higher among male household contacts (22.4%) than among females (17.9%) (p = 0.003) [49], so the evidence for an effect of gender on seropositivity levels is not consistent.

### The effect of type of test on seroprevalence

In total six types of serological assays were identified, all based on detection of antibodies against the *M*. *leprae*-specific PGL-I or derivatives thereof. All papers reported measurement of the IgM subtype, probably because anti-PGL-I IgM seroprevalence is higher than IgG/IgA seroprevalence in endemic areas. Moreover, IgM seropositive individuals have a higher risk of developing disease [80]. However, IgM seropositivity is not predictive for disease [56,60], whereas anti-PGL-I IgG has been described to predict leprosy disease [56]. However, to assess (past) infection with *M*. *leprae* as a proxy for transmission, anti-PGL-I IgM is a much more sensitive biomarker than anti-PGL-I IgG as it can be detected in many individuals (previously) infected with the mycobacterium despite the absence of disease [40,71,72,80].

Three individual studies each using a single, study-specific test showed that anti-PGL-I seropositivity in children correlated with the level of leprosy endemicity [35,65,76]: in India seropositivity rates of 0%, 6.9% and 21.4% were described for a reportedly low-endemic region, a high-endemic region and a contact population, respectively [65]. In Sulawesi [35] seropositivity rates ranging from 26 to 28% were measured in three reportedly high-endemic areas (case detection rates > 30/100,000) compared to 7% in two low-endemic areas (case detection rates of 10/100,000). Of note was that seroprevalence and incidence rates as well as their correlation remained stable over a period of three years. In 2020, an Indonesian study reported seropositivity of 8.3% in Pacitan (considered a non-endemic district) compared to 48% in Lamongan (considered an endemic district) [76]. Thus, these studies convincingly show the relation between the overall disease incidence in an area and the seroprevalence among children. On the other hand, in one study among 7,073 schoolchildren in South(east) Brazil [36], seropositivity levels (ranging from 8.5 to 24%) did not correlate completely with endemicity (ranging from 2 to 112/100,000 population). The authors suggest that this may have been caused by differences in immune responsiveness per population, e.g. different social economic status, which may cause differences in disease incidence whereas asymptomatic *M*. *leprae* infection may be common. Alternatively, case detection depends on operational factors, such as local health structure, active case finding and the local ability to diagnose leprosy cases which may lead to underdiagnosis in certain areas. This may be an additional advantage of monitoring serology in children. Also, the authors mentioned that the study sample may not always have been representative for the total population in certain municipalities as participation was dependent on the presence of schoolchildren at school at the time of study and consent from their parents. Nevertheless, the seropositivity percentages reported seem reliable, as most of them lie in the same range as the six other studies from the same area discussed above [47–52].

Direct comparison of results between studies was difficult due to the variety of tests and/or different cut-off levels used. For instance, measurements were performed using either fingerprick blood or serum with variable dilutions, and the target antigens in the tests varied from native PGL-I to synthetic PGL-I recognized by various IgM, IgG or IgA isotypes. Importantly, cut-off values were often chosen arbitrarily. For example, in the state of Pará in Brazil, the PGL-I IgM ELISA data of Barreto *et al*. [42] and van Hooij *et al*. [28] for a selection of 58 schoolchildren from Pará correlated excellently (S2 Fig), but there was a large disparity in seropositivity rates because of differences in determination of the cut-off value for positivity. This stresses that the test cut-off used is vital and may impede comparison of seroprevalence data between studies even if quantitative levels correlate well.

The type of serological test and standardisation of the cut-off for positivity used in seroscreening for assessment of transmission is thus crucial. In addition, quantitative results (being either obtained by ELISA or field-friendly lateral flow tests) can potentially assess differences in seropositivity with higher accuracy than operator-dependent, visual tests, which is beneficial for monitoring changes in transmission over time within an area and for assessing seroprevalence in very low-endemic areas. Since leprosy-endemic areas are often situated in low resource settings, field-friendly rapid tests are recommended. In this review three types of field-friendly tests were identified, in two of which data were obtained visually and scored qualitatively by operators after five to ten minutes [49,50,55], whereas the anti-PGL-I UCP-LFA [28] offers quantitative determination of antibodies. Of note is that anti-PGL-I IgM UCP-LFA provided similar sensitivity and specificity as the NDO-LID rapid-test [28] and can also be used for and/or in combination with different biomarkers (multiplexing) thereby offering added value [45,81].

### Limitations

No formal methods for assessing risk of bias (a component of PRISMA) were used due to the variation of study methods in the selected studies. The fact that we eventually have included English, Spanish and Portuguese articles only in our literature search may have slightly limited the completeness of this overview. However, these are the languages in which studies of the two most endemic countries are reported. Our strategy resulted in the exclusion of eight articles in French, three in Japanese, one in Swedish and one in Bahasa, Indonesia that were identified through Pubmed, Infolep and Web of Science of which only the latter article [82] seemed eligible for inclusion as the other papers did not describe leprosy serology in children (based on screening of the English abstracts). Furthermore, the variety of serological tests used in the selected studies impede direct comparison of the results.

## Conclusions

On the route to leprosy elimination, an indicator for the intensity of transmission would be highly valuable. Quantitative anti-PGL-I serology in young children as a measure for *M*. *leprae* infection shows potential as a proxy for recent transmission and also correlates with disease incidence. Therefore, it can provide a means to monitor progress towards interruption of transmission, as well as to assess the effect of prophylactic interventions on transmission. This systematic review also underscores that, to collect definitive evidence on the utility of PGL-I serology in (young) children as a marker of transmission of *M*. *leprae*, further studies are needed to evaluate a standardized, quantitative test in multiple areas with different levels of leprosy endemicity. In addition, to assess transmission in an area over time, cross-sectional studies on individuals of the same age group and not on the same individuals are needed. In such studies, the target group would be children without leprosy who are not known contacts of (former) leprosy patients. Based on the findings of this literature search, primary school-age children would represent a suitable target group for sensitive detection of recent transmission, which will allow monitoring of its elimination.

## Supporting information

S1 Table Research PRISMA Checklist–Detection of anti-*M*. *leprae* antibodies in children in leprosy-endemic areas: A systematic review. Research PRISMA checklist. ^1^ No formal methods for assessing risk of bias were used due to the variation of study methods. Study limitations influencing outcomes and conclusions were considered and described in the Discussion section of the review.
(DOCX)

S2 Table Seroprevalence results for *M*. *leprae* antibodies in children without leprosy sorted per country. Overview per country of the information gathered from the studies included in this review reporting data on serology for *M*. *leprae*-specific antigens. For each study, the country/area, reference number, author, year of publication, test group, age (range), number of children, prevalence of seropositivity (%) for *M*. *leprae* antibodies, sample, test target and laboratory assay are reported. *Estimated from graph/figure. Ab: antibody; CP: contact population from a leprosy colony; ELISA: enzyme-linked immunosorbent assay; F: females; GPAT: gelatin particle agglutination test; HER: children from reported high-endemic region; IgA: immunoglobulin A; IgG: immunoglobulin G; IgM: immunoglobulin M; LER: children from reported low-endemic region; LID-1: leprosy IDRI diagnostic-1; M: males; (N)D-O-BSA: (natural) disaccharide-octyl bovine serum albumin; ND-O-HSA: natural disaccharide-octyl human serum albumin; ND-O-LID: semi-synthetic disaccharide attached to the octyl radical, which mimics PGL-I, conjugated with two fusion proteins, ML0304 and ML0331, forming LID; NTO: natural trisaccharide-octyl; NT-P-BSA: natural trisaccharide phenylpropionyl bovine serum albumin; PGL-I: phenolic glycolipid-I; ref. no.: reference number; UCP-LFA: up-converting phosphor lateral flow assay.
(XLSX)

S3 Table Test methods applied for detection of antibodies against *M*. *leprae*-specific antigens. Overview of the details of the methods used in the studies (n = 30) reporting serology data on *M*. *leprae*-specific antibodies in children. For each study, the reference number, author, year of publication, country, sample, test target, nature of the test target, antibody isotype, assay, readout, dilution, cut-off and cut-off explanation are listed. Ab: antibody; CP: contact population from a leprosy colony; ELISA: enzyme-linked immunosorbent assay; F: females; GPAT: gelatin particle agglutination test; HER: children from reported high-endemic region; IgA: immunoglobulin A; IgG: immunoglobulin G; IgM: immunoglobulin M; LER: children from reported low-endemic region; LID-1: leprosy IDRI diagnostic-1; M: males; (N)D-O-BSA: (natural) disaccharide-octyl bovine serum albumin; ND-O-HSA: natural disaccharide-octyl human serum albumin; ND-O-LID: semi-synthetic disaccharide attached to the octyl radical, which mimics PGL-I, conjugated with two fusion proteins, ML0304 and ML0331, forming LID; NTO: natural trisaccharide-octyl; NT-P-BSA: natural trisaccharide phenylpropionyl bovine serum albumin; OD: optical density; PGL-I: phenolic glycolipid-I; ref. no.: reference number; UCP-LFA: up-converting phosphor lateral flow assay.
(XLSX)

S4 Table Longitudinal studies included in the review. Results of the longitudinal studies included in the review. For each study, the reference number, the author, year of publication, country (area), test group, age in years, number of participants, follow-up period, type of follow-up, seropositivity and development of disease results are shown (when available). IgA: immunoglobulin A; IgG: immunoglobulin G; IgM: immunoglobulin M; sero+: seropositive; sero-: seronegative.
(XLSX)

S1 Fig Overview of seropositivity in different study areas. Overview per area of the serology data for *M*. *leprae*-specific antigens gathered from the studies included in this review. Each dot represents one of the seropositivity percentages reported. The horizontal lines represent the median of the results found in a specific area. A: Seropositivity data reported among children who were not known to be contacts of leprosy patients. B: Seropositivity data reported among children known to be contacts of leprosy patients. C: Boxplot of all the seropositivity data gathered. Min: minimum; max: maximum; Q1: first quartile; Q3: third quartile. Contacts represent children living in the household/direct vicinity or neighborhood of leprosy patients; non-contacts represent children without known contact to leprosy patients. In case multiple seropositivity percentages were reported in one article, those were included in line with how they were presented e.g., per district, per age group, per test and depicted separately.
(TIF)

S2 Fig Correlation analysis of ELISA data from different studies. A: Correlation of the anti-PGL-I IgM ELISA data of sera of children (n = 58) below the age of 15, assessed as described in Barreto *et al*. [1] and van Hooij *et al*. [2]. The latter adjusted results for background OD for each sample. Data for the same samples correlated excellently (p<0.0001; Pearson r = 0.92). The red dotted line represents the cut-off value (OD_450_: 0.295) applied in Barreto *et al*. [1]; the blue dashed line represents the cut-off value (OD_450_: 0.2) applied in van Hooij *et al*. [2]; the black box indicates the twenty samples with values around the cut-off value; the samples with green border color scored positive as analyzed by Barreto *et al*. [1]. B: Twenty samples with values around the cut-off (as indicated by the black box in A) as analyzed by Barreto *et al*. ([1]: OD_450_ ranging from 0.274 to 0.312; cut-off OD_450_: 0.295) and van Hooij *et al*. ([2]: OD_450_ corrected for background ranging from 0.043 to 0.312; cut-off OD_450_: 0.2). The samples with green border color scored positive as analyzed by Barreto *et al*. [1]. C: Selection of the nine samples around the cut-off value that scored positive (green border color in A and B) as analyzed by Barreto *et al*. ([1]: OD_450_ ranging from 0.297 to 0.312; cut-off OD_450_: 0.295) and van Hooij *et al*. ([2]: OD_450_ corrected for background ranging from 0.063 to 0.308; cut-off OD_450_: 0.2).
(TIF)
