# Supplementary figures and images for: Detection of anti-M. leprae antibodies in children in leprosy-endemic areas: A systematic review
Source: PLoS Negl Trop Dis. 2021 Aug 27;15(8):e0009667. doi: 10.1371/journal.pntd.0009667 (PMC8428563; doi:10.1371/journal.pntd.0009667)

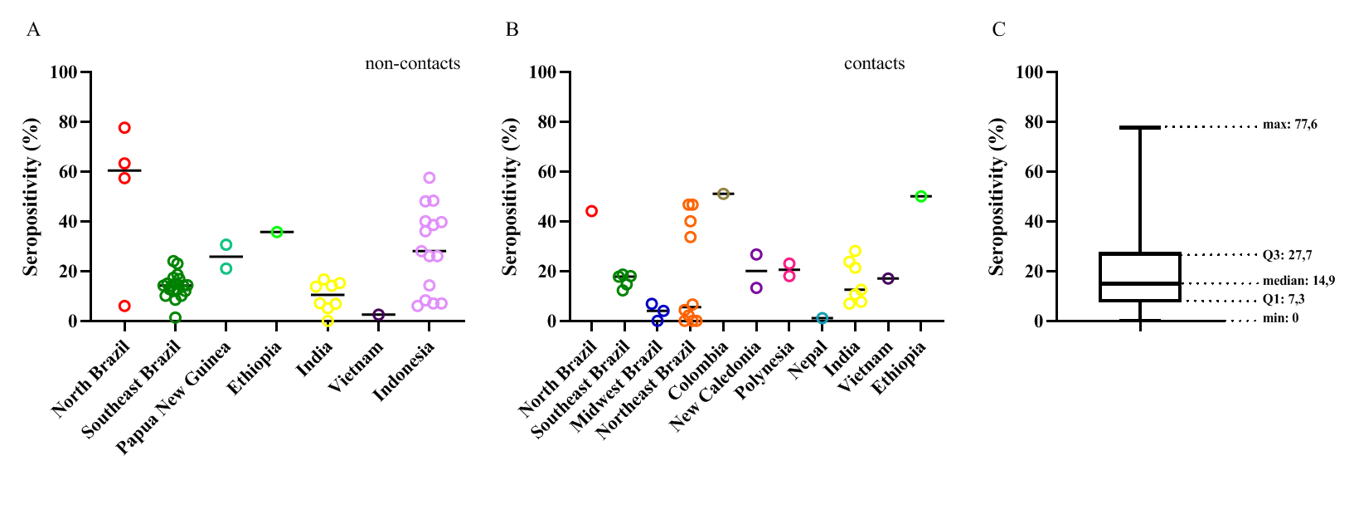

Supplement: S1 Fig — Overview per area of the serology data for M. leprae-specific antigens gathered from the studies included in this review. Each dot represents one of the seropositivity percentages reported. The horizontal lines represent the median of the results found in a specific area. A: Seropositivity data reported among children who were not known to be contacts of leprosy patients. B: Seropositivity data reported among children known to be contacts of leprosy patients. C: Boxplot of all the seropositivity data gathered. Min: minimum; max: maximum; Q1: first quartile; Q3: third quartile. Contacts represent children living in the household/direct vicinity or neighborhood of leprosy patients; non-contacts represent children without known contact to leprosy patients. In case multiple seropositivity percentages were reported in one article, those were included in line with how they were presented e.g., per district, per age group, per test and depicted separately. (TIF) [file pntd.0009667.s005.tif]

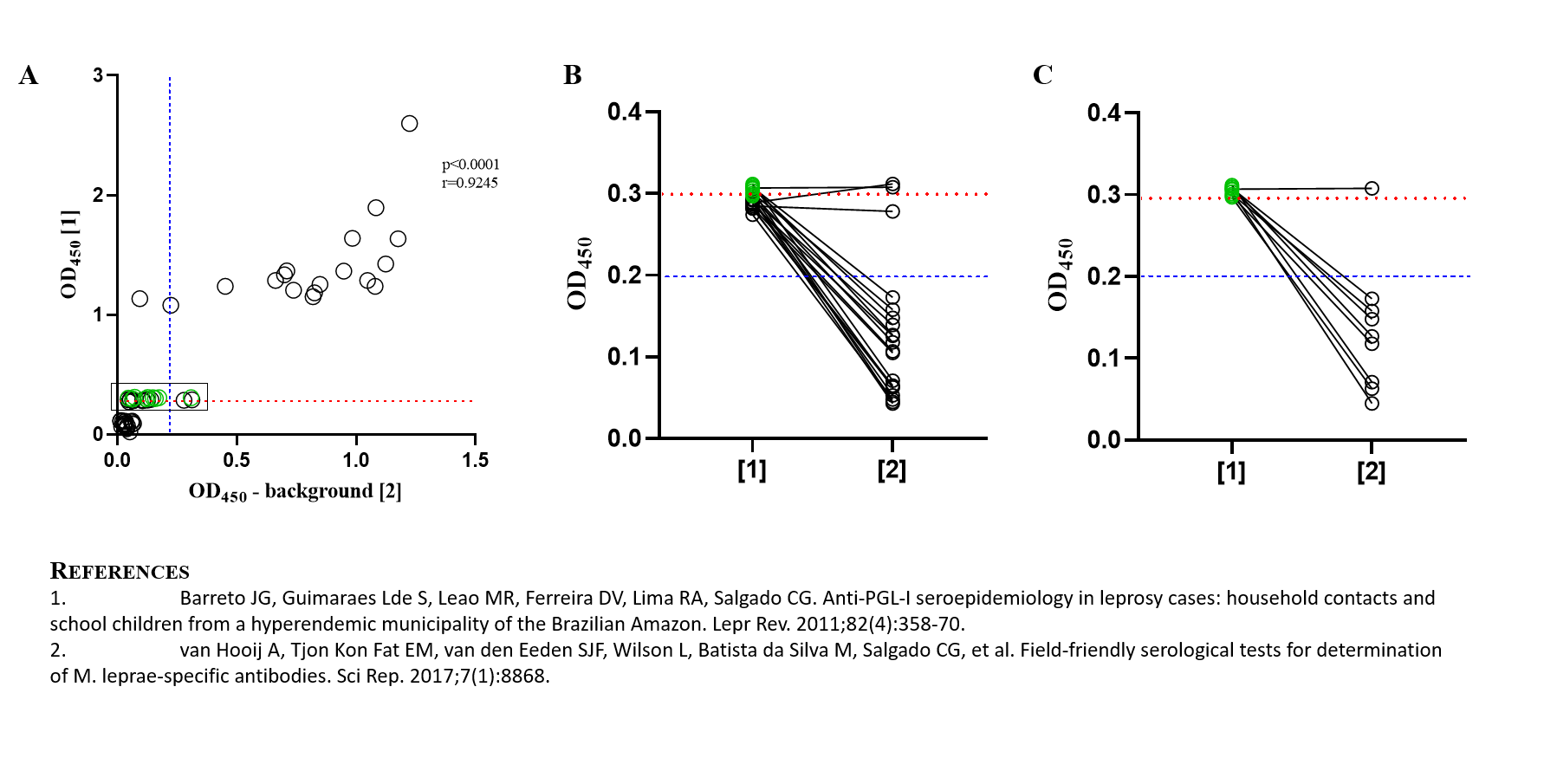

Supplement: S2 Fig — A: Correlation of the anti-PGL-I IgM ELISA data of sera of children (n = 58) below the age of 15, assessed as described in Barreto et al. [1] and van Hooij et al. [2]. The latter adjusted results for background OD for each sample. Data for the same samples correlated excellently (p<0.0001; Pearson r = 0.92). The red dotted line represents the cut-off value (OD450: 0.295) applied in Barreto et al. [1]; the blue dashed line represents the cut-off value (OD450: 0.2) applied in van Hooij et al. [2]; the black box indicates the twenty samples with values around the cut-off value; the samples with green border color scored positive as analyzed by Barreto et al. [1]. B: Twenty samples with values around the cut-off (as indicated by the black box in A) as analyzed by Barreto et al. ([1]: OD450 ranging from 0.274 to 0.312; cut-off OD450: 0.295) and van Hooij et al. ([2]: OD450 corrected for background ranging from 0.043 to 0.312; cut-off OD450: 0.2). The samples with green border color scored positive as analyzed by Barreto et al. [1]. C: Selection of the nine samples around the cut-off value that scored positive (green border color in A and B) as analyzed by Barreto et al. ([1]: OD450 ranging from 0.297 to 0.312; cut-off OD450: 0.295) and van Hooij et al. ([2]: OD450 corrected for background ranging from 0.063 to 0.308; cut-off OD450: 0.2). (TIF) [file pntd.0009667.s006.tif]
